# High performance team-based care for persons with chronic conditions

**DOI:** 10.1186/s13584-015-0003-1

**Published:** 2015-02-19

**Authors:** Stephen C Schoenbaum, Sally Okun

**Affiliations:** Josiah Macy Jr. Foundation, 44 East 64th Street, New York, NY 10065 USA; PatientsLikeMe, 155 Second Street, Cambridge, MA 02141 USA

## Abstract

Care for patients with complex chronic conditions such as diabetes requires a coordinated and collaborative team working in partnership with the patient. Israel has taken important steps forward with the development of structured diabetes follow-up by Clalit Health Services, including several measures of diabetes care in the National Program for Quality Indicators in Community Healthcare, and efforts to develop health information exchange and measures of continuity between hospital and community-based care. Achieving even better results will require purposeful development of health care teams to meet the needs of patients with single and multiple chronic conditions, including robust interprofessional education programs for the next generation of health professionals, and developing partnerships between the teams and the patients.

## Commentary

Diabetes is a chronic condition that is characterized by high blood sugar levels when untreated or inadequately treated. It is a complex condition that can, and often does, involve the kidneys, eyes, nervous system, heart, and blood vessels. Comprehensive clinical expertise and self-management skills are needed to support physical, mental, and social wellbeing of diabetics; and integrating high quality diabetes care into the primary care setting requires a coordinated and collaborative team working in partnership with the patient.

The findings in a recently published paper in this journal about the structured diabetes follow-up protocol introduced by Clalit Health Services in 1995 [1], and the findings in the chapter on the quality of diabetes care in the 2012 OECD review of health care quality in Israel [2], provide strong evidence that people living with diabetes can experience improved clinical outcomes when care is provided in a locally organized team-based primary care environment highlighted by dedicated nurse coordinators.

The Clalit *Diabetes in the Community* program was for its time an important step. It aimed to improve follow-up for diabetics through 2–4 visits per year with clinic nurses. The visits included education related to diabetes management. The program considered complete diabetes follow-up to include: HbA1c, LDL cholesterol, microalbumin, blood pressure measurements, and fundus examination. Today we know that follow-up should be even broader and include items specifically mentioned in the OECD report such as monitoring performance on foot examinations and screening for mental health issues.

The OECD report further states that lower extremity amputation rates in Israel are relatively high (16.9 per 100,000 population) compared to other OECD countries (average = 11.4 per 100,000 population).

Fortunately, some of the health plans already have put in place internal quality measures related to depression and to foot care; and the National Program for Quality Indicators in Community Healthcare in Israel (QICH) is also looking into the adoption of such measures. One would hope that lower extremity amputation rates would improve once the metric is in place and there is a focus on diabetic foot care.

Integrating mental health and social care screening should be considered as an opportunity to further support the health and wellbeing of persons with diabetes. Despite the fact that the list of primary care data collection measures in the QICH is considered “one of the most impressive examples of primary care data collection among OECD countries” [2] there are no measures of mental or social issues included for any of the six key topic areas covered in the QICH, including diabetes.

Screening, an important first step, helps identify persons who can benefit from targeted preventive and therapeutic services; and analysis of screening and process data across populations helps in developing targeted programs. Diabetes, for instance, is more prevalent among Arab Israelis [2]; and as shown in an article in this journal from Maccabi Healthcare Services, multiple chronic diseases are more prevalent among older and poorer Israelis [3]. Programs at the community and practice level are, and in the future should be targeted to, and tailored for, high risk groups.

In the 21^st^ century, there is a high and increasing prevalence of single and multiple chronic conditions in the populations of developed countries, including Israel [3]. It has been known for many years that patients with multiple chronic conditions account for a very large percentage of total health care costs [4], estimated to be as high as 75 percent of total costs in the United States (U.S.). It is extremely important that health care providers work together in multi-disciplinary, interprofessional teams with patients to manage the conditions more effectively and efficiently and achieve better outcomes [5].

As described by Mitchell et al. in a discussion paper from the Institute of Medicine in the U. S. (IOM), implementing effective team-based care requires intentional integration of key core values and principles that can be measured and compared for learning and replication [6]. To realize the full potential that team care offers, investments will be needed by individuals, institutions, organizations, and governments. A subsequent discussion paper from the IOM on fostering effective partnerships with patients and healthcare teams presents insights gleaned directly from patients and providers about their experiences with team-care in a variety of primary care settings [7]. Although not included in the final paper, the study group discussed at length a set of essential characteristics (each beginning in English with the letter “C”) that are needed for effective, high quality team-based care. These are:

- Coordination—No matter the size or type of team, its component parts need to coordinate and it needs to have a mechanism for effectively resolving conflicting points of view.
- Collaboration—All team members, including patients and family care givers, must work together, including resolution of differences of opinions.
- Communication—Without effective two-way communication, there can be no collaboration. Essential communication skills include the ability, cultural sensitivity, and competence to work effectively with diverse team members and patients.
- Continuity—When teams are comprised of many different health professionals, continuity of care means ensuring patients that they have reliable access to one or two key individuals.
- Connectedness—Care that begins in one setting frequently continues in a different setting and at the patient’s home. For patients with chronic health conditions or multiple ailments, care can occur at multiple sites, meaning the patient’s health care team can expand beyond any single team providing care at any individual facility. These settings must be connected, especially with updated information relevant to the patient.
- Comprehensiveness—Patients and family caregivers frequently lament that their care addresses their current health condition, rather than treating them as a “whole person.” Effective teams will recognize the implications of a given issue and suggest, recommend, and facilitate connection with other professionals who can assist with addressing the patient’s other needs.
- Compassion—It is necessary to acknowledge and validate concerns, pain, distress, suffering; respond with empathy, care and concern; and work together to try to ameliorate these conditions.
- Continuous improvement –Assessment of team performance, both process and outcome; learning; and improvement must occur continuously.

Much remains to be done in Israel and elsewhere to develop high-functioning health care teams for patients with chronic conditions such as diabetes and to develop effective partnerships with patients. In addition, the 2012 OECD report specifically indicates that there needs to be better coordination of care in Israel between the hospital and ambulatory/community-based settings. Recent efforts to improve such coordination are promising, including work on the development and deployment of a national mechanism for exchanging health information; an effort to develop indicators of continuity between hospitals and the community; and the German Committee’s recommendations of practical measures to improve care coordination.

Not only do current health professionals need to work increasingly well in interprofessional teams, but also the next generation of health professionals must be prepared to function effectively in a variety of teams and to know how to establish and maintain partnerships with patients. This is the rationale for interprofessional education (IPE) which is defined by the World Health Organization as occurring “when students from two or more professions learn about, from and with each other to enable effective collaboration and improve health outcomes” [8]. In Israel, we believe that only Ben-Gurion University currently has a formal IPE curriculum for undergraduate students in various health professions. Most persons involved in IPE believe it is important that students be developing their interprofessional identity at the same time as their professional identity. A main reason for starting early is to deal with any pre-existing biases as learners enter school and to minimize the development of the biases and stereotypes that are known to be quite strong when health professionals enter practice. Teams are strongest when they are composed of people with very different strengths/identities who have learned how to take advantage of the strengths of each other member of the team. Having a strong professional identity is important; and knowing how to work well with others with different identities is important. Achieving excellent interprofessional collaboration requires constant effort, and this should continue throughout the postgraduate training and careers of health professionals. As interprofessional education programs are extended throughout the country and across as many health professions as possible, in the future there should be more effective and efficient health care teams in and across all settings.

In the specific instance of providing care to persons with complex chronic conditions such as diabetes and persons with multiple chronic conditions, it is a *sine qua non* that health care teams must function well internally to be able to establish effective partnerships with patients. The team and its members must value the mutual interactions with patients and must welcome them as partners in achieving excellent health outcomes. Since patients live with their conditions all the time, they are the experts on how they feel. Most are capable of mastering the knowledge and skills necessary to manage most of the demands of their conditions. But, none, even those who are health professionals themselves, have mastered the knowledge and skills of *all* of the health professions. Patients can contribute something important to their health care teams, but they also have needs that must be met by the team and its members. For the partnership to function well, the patient must have clarity about roles and know how to access reliably the appropriate member(s) of the team. Now that there is structured follow-up for diabetic patients in many primary care settings in Israel, it will be important as a next step to structure the way patients can access their information and their health care provider teams when necessary.

The OECD, in summarizing its comments about quality of care in Israel reports, “Over the past decade and a half, policy makers and health plans have sought to reorganise doctors working in the community into teams. This has provided them with a platform to do things that other OECD countries are struggling to do, like regular monitoring of a patient’s health indicators, delivering follow-up support after a visit to the doctor, and tailoring preventative advice to the specific needs of communities.” Indeed, this is an excellent platform to build upon to address some of the challenges that we have pointed out above. It should be possible for Israel, based on Clalit’s experience with structured nursing follow-up for diabetics [1]; the QICH with its quality measures for asthma, cancer screening, child and adolescent health, immunization for older adults, cardiovascular health, as well as diabetes; and the information on prevalence of chronic conditions reported from Maccabi [3], to develop better team care and partnerships with patients who have a variety of chronic conditions, especially those patients who have multiple chronic conditions.
